# Author Correction: Neurons and neuronal activity control gene expression in astrocytes to regulate their development and metabolism

**DOI:** 10.1038/ncomms16176

**Published:** 2018-02-06

**Authors:** Philip Hasel, Owen Dando, Zoeb Jiwaji, Paul Baxter, Alison C. Todd, Samuel Heron, Nóra M. Márkus, Jamie McQueen, David W. Hampton, Megan Torvell, Sachin S. Tiwari, Sean McKay, Abel Eraso-Pichot, Antonio Zorzano, Roser Masgrau, Elena Galea, Siddharthan Chandran, David J. A. Wyllie, T. Ian Simpson, Giles E. Hardingham

Nature Communications 8: Article number: 15132; DOI: 10.1038/ncomms15132 (2017); Published: 05 02 2017; Updated: 02 06 2018

Michel Goedert, who developed the Thy1-P301S transgenic mouse, was inadvertently omitted from the Acknowledgments section of this Article. The Acknowledgements should have included the following:

‘We thank Michel Goedert for providing the Thy1-P301S transgenic mouse that was used in this study.’

