# Supplementary material for: Neurons and neuronal activity control gene expression in astrocytes to regulate their development and metabolism
Source: Nat Commun. 2017 May 2;8:15132. doi: 10.1038/ncomms15132 (PMC5418577; doi:10.1038/ncomms15132)
Supplement: Supplementary Information — Supplementary Figures. [file ncomms15132-s1.pdf]

Supplementary Figures

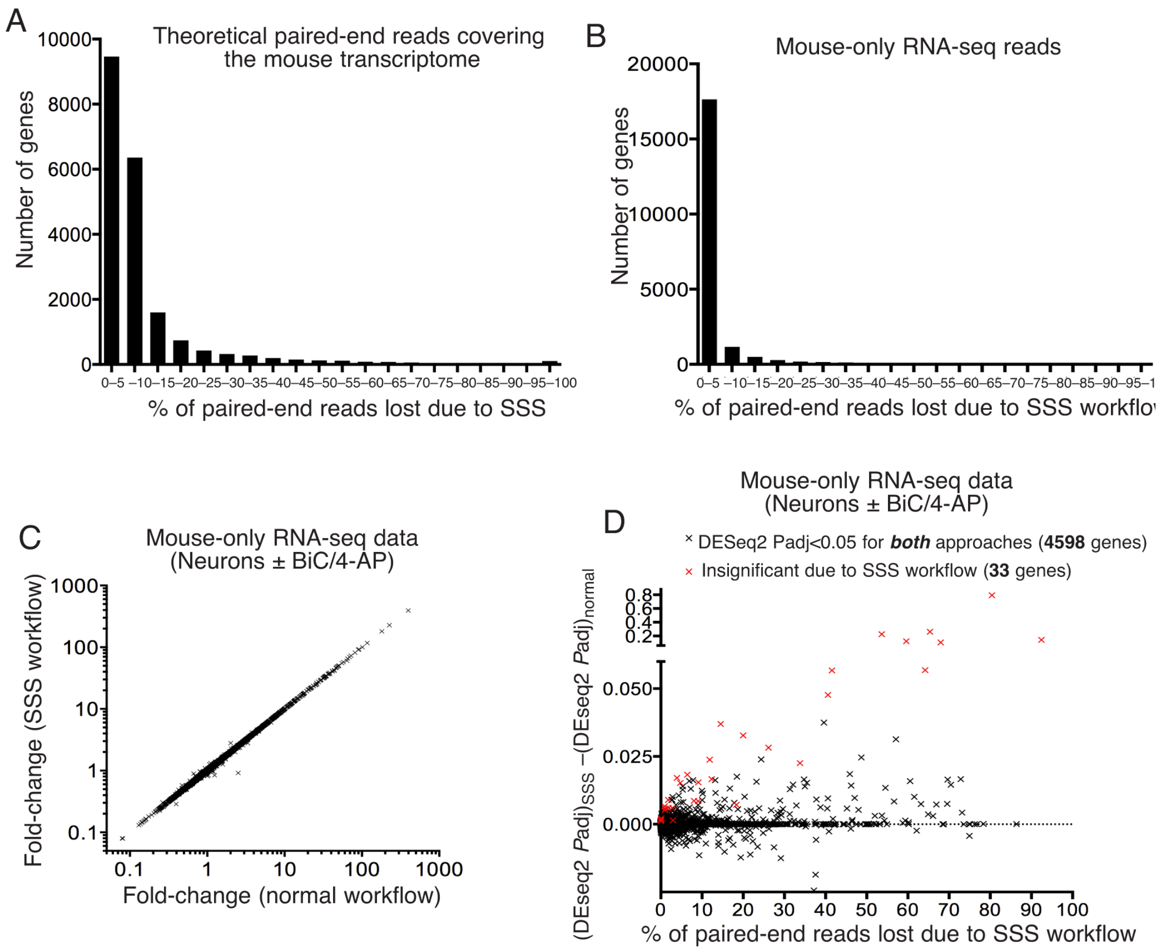

Supplementary Figure 1. Feasibility of species-specific sorting of mouse RNA-seq reads away from rat reads. A) From the mouse protein-coding transcriptome (minus predicted genes) in Ensembl version 82, all possible theoretical 50 nt paired-end reads, with insert size 150 nt were generated, and subjected to both the SSS-workflow and conventional STAR mapping. The % of reads lost due to SSS was calculated on a gene-by-gene basis, and a frequency distribution histogram (5% bins) generated. NB. only a single nucleotide difference across both 50 nt windows is required for correct species attribution**.** See Supplementary Data 1. **B)** A small % of RNA-seq reads are lost due to SSS. RNA-seq reads were generated from RNA extracted from a pure mouse neuronal culture (n=3). The paired-end reads were subjected to both the SSS-workflow and conventional STAR mapping, with both approaches requiring full-length reads with no mis-matches, and the former requiring non-ambiguity of species. The % of reads lost due to SSS (compared to conventional mapping) was calculated on a gene-by-gene basis, for all protein-coding genes (except predicted genes) and a frequency distribution histogram (5% bins) generated. See Supplementary Data 2. **C)** Gene fold-change is little-altered by species-specific sorting of RNA-seq reads. Pure neuronal mouse cultures were treated ± BiC/4-AP for 4h (BiC: 50 µM, 4-AP: 250 µM), RNA extracted and RNA-seq performed (n=3). We then performed differential gene expression (DGE) analysis on the data set, using both SSS and normal approaches, to identify genes induced or repressed by BiC/4-AP-induced synaptic activity, focussing on the 11,059 genes whose expression was >2 FPKM. The fold-changes derived from the different mapping methods were plotted against each other. All data are available in Supplementary Data 3. **D)** Read losses due to SSS have little impact on *P*-value significance. 4631 genes were called as being significantly up- or down regulated by BiC/4-AP treatment (DESeq2 *P*_adj<0.05) using the normal STAR mapping method. For those same genes, the DESeq2 *P*_adj was calculated after the SSS-workflow, and the increase in *P*-value calculated. This increase was plotted against the % of reads lost due to the SSS-workflow.

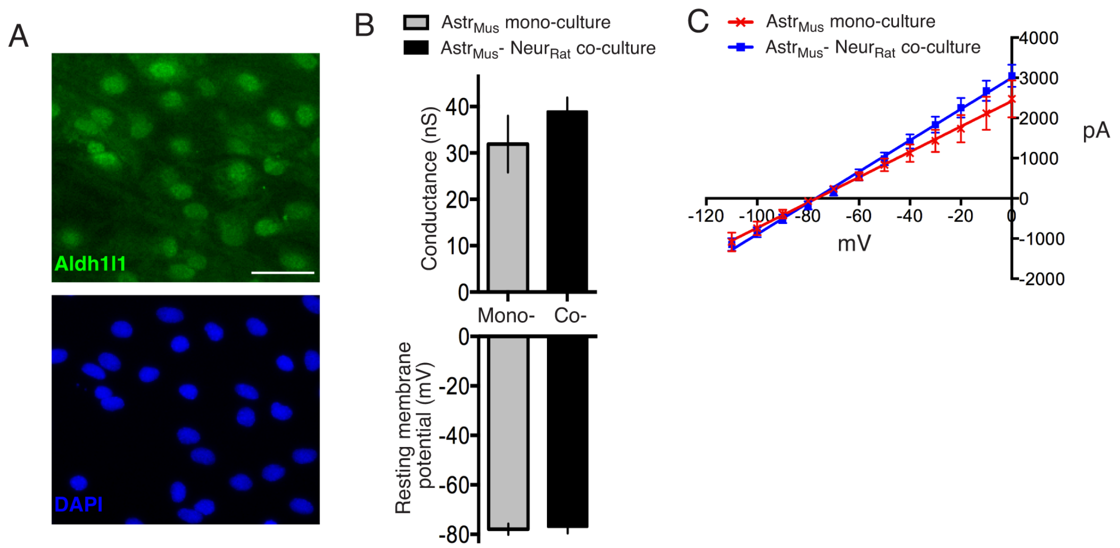

Supplementary Figure 2. A) Immunofluorescent staining of astrocyte cultures with an anti-Aldh1l1 antibody (upper) or DAPI (lower, scale bar: 50 µm). **B,C)** Passive membrane IV-relationship in astrocytes is not affected by co-culturing with neurons. IV relationship (**C**) and slope (**B, upper**) are shown. n=8 mono-cultured n=9 co-cultured astrocytes. For these cells, the resting membrane potential was also measured (**B, lower**).

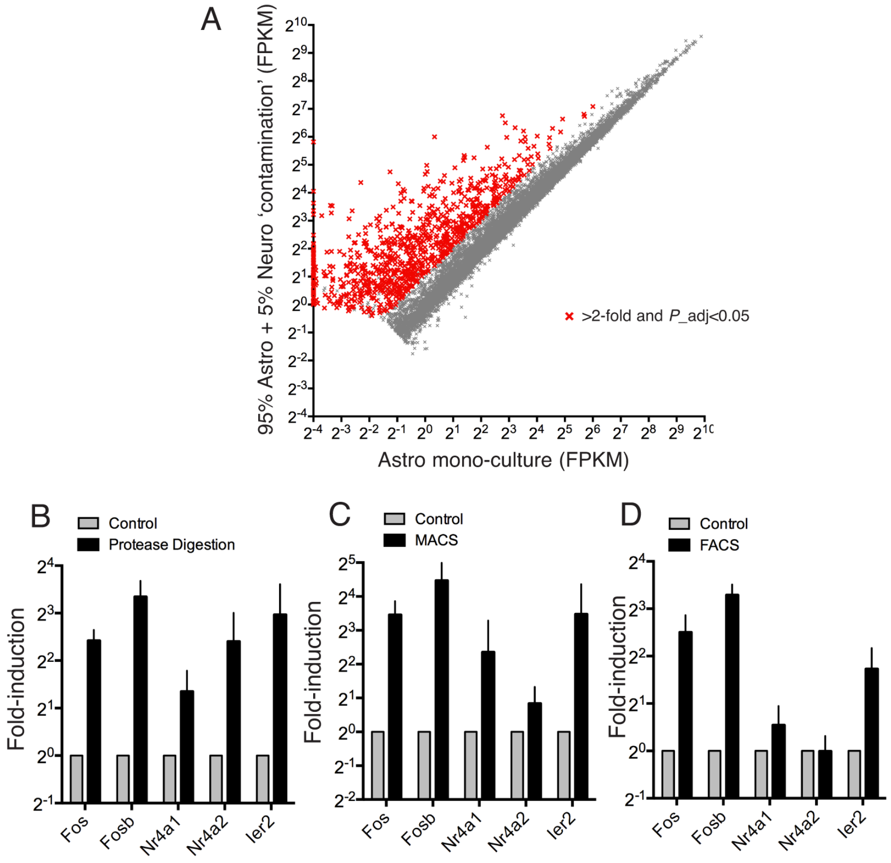

Supplementary Figure 3. A) A small degree of cell-type contamination can lead to false positives when studying gene regulation. A sorting process was simulated to achieve 95% purity, by 'contaminating' the mouse astrocytic mRNA with mouse cortical neuronal mRNA at a ratio of 95:5, by number of cells harvested. RNA-seq was performed on the pure astrocytic mRNA and the 95:5 astrocytic/neuronal mRNA (n=3 biological replicates). Red stars indicate genes significantly altered >2-fold (P_adj<0.05) as a result of the contamination (also see Supplementary Data 7). **B,C,D)** Physical sorting and cell dissociation protocols induce transcriptional responses. B) A pure mono-culture of astrocytes was digested with trypsin to generate a single cell suspension, after which cells were spun down, snap frozen, and RNA subsequently extracted and the indicated genes analysed by qPCR (normalised to *H1f0*, n=3 biological replicates). C) As for (B) except that after trypsinization the cells were subjected to a MACS astrocyte immunopanning protocol prior to RNA extraction. D) As for (B) except that after trypsinization the cells were put through a FACS sorter (ungated) prior to RNA extraction.

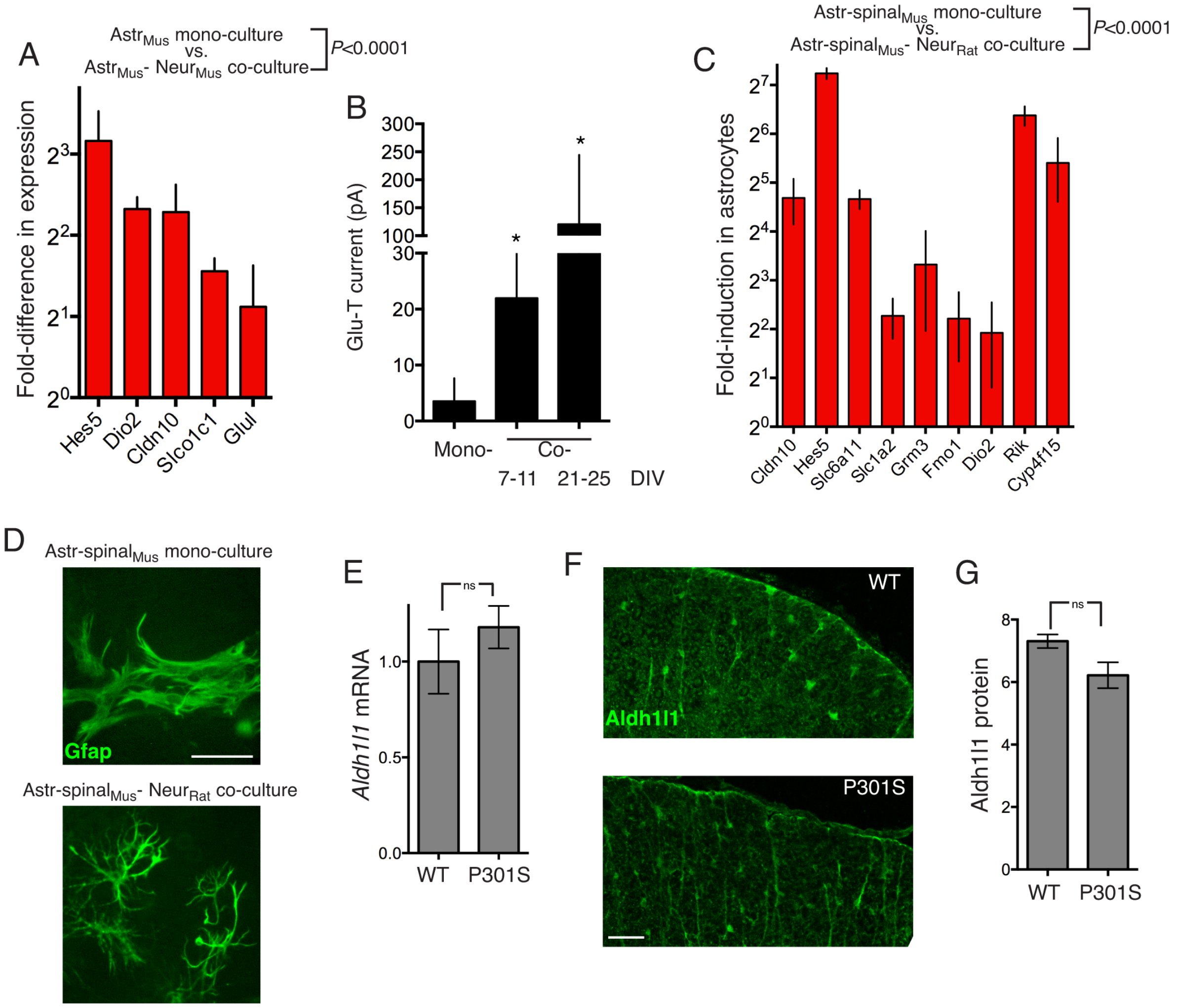

Supplementary Figure 4. A) Expression of the indicated genes was analysed in mouse astrocytic cultures and mixed mouse astrocyte/neuronal cultures, normalized to H1f0. A 2-way ANOVA was performed and the main effect of culture type reported (P<0.0001, n=4). **B)** Glutamate transporter currents in response to 200 µM L-Aspartate application were measured in astrocytes in either pure astrocyte cultures (mono-) or mixed astrocyte/neuronal (co-) cultures of the indicated ages. *P<0.05, t-test relative to mono-culture (n=16 cells (mono), 21 cells (Co- DIV7-11), 10 cells (DIV21-25)) **C)** Expression of the indicated genes was analysed in spinal cord astrocytes in the presence or absence of neurons (co-cultured for 9 days). P<0.0001 (main effect of culture type, 2-way ANOVA, n=3). **D)** Example images of Gfap-stained spinal cord astrocytes cultured in the absence (upper) or presence (lower) of neurons (scale bar: 100 µm). **E)** Expression of *Aldh1l1* mRNA (n=6 per genotype) was calculated by generating cDNA from identical amounts of total RNA, performing qPCR using Aldh1l1 primers, and noting the threshold cycle number (Ct) at which the amplification becomes exponential. Concentration of *Aldh1l1* mRNA was calculated as $2^{\wedge -Ct}$. **F)** Example micrographs of Aldh1l1 immunofluorescence in spinal cord sections of WT and P301S genotypes. **G)** Quantification of Aldh1l1 immunofluorescence (n=4 animals per condition; 12-15 images per animal). Scale bar: 100 µm.

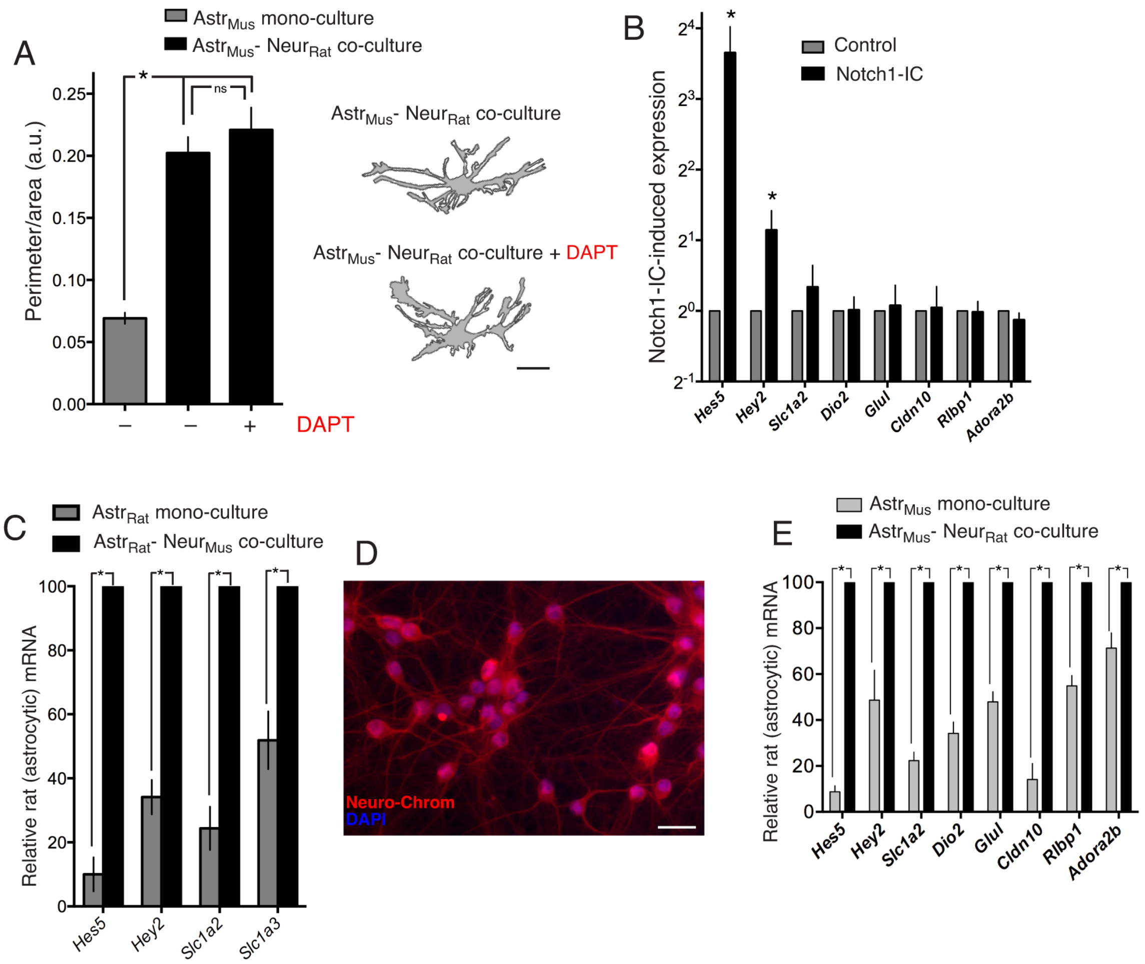

Supplementary Figure 5. A) Mouse astrocytes were transfected with GFP before being overlaid where indicated with rat neurons in the presence or absence of DAPT. The cells were fixed and morphology assessed after 9 days as described in Fig. 1b. * P<0.05, 1-way ANOVA plus Tukey's post-hoc test (n=20 of each condition). Scale bar: 20 µm. **B)** Mono-cultures of astrocytes were transfected with a control vector or one encoding Notch1-IC. The indicated genes were analysed 48h later by qPCR. *P<0.05, t-test (n=5). **C)** Swapping the species of neurons and astrocytes (i.e. $Astr_{Rat}$- $Neur_{Mus}$ co-cultures, instead of $Astr_{Mus}$- $Neur_{Rat}$ co-cultures) does not alter neuron-to-astrocyte gene expression. Expression of Notch target genes *Hes5* and *Hey2*, and glutamate transporter genes *Slc1a2* and *Slc1a3*, were studied using rat-specific qPCR primers in rat astrocytes cultured in the presence or absence of mouse neurons. *P<0.05 (1-way ANOVA plus Sidak's post-hoc test, n=4). **D)** Neuro-Chrom staining of DIV9 cortical neurons cultured in the presence of AraC (>99% Neuro-$Chrom^+$). Scale bar: 20 µm. **E)** Mouse astrocytes were cultured in the presence or absence of mouse neurons, with AraC added at the same time as the neuronal culture. RNA-seq was performed and the SSS-workflow applied followed by DGE analysis of the mapped mouse reads (i.e. astrocytic transcripts). The data shown relates to the genes studied in Fig. 4c (**P*_adj<0.05, n=3)

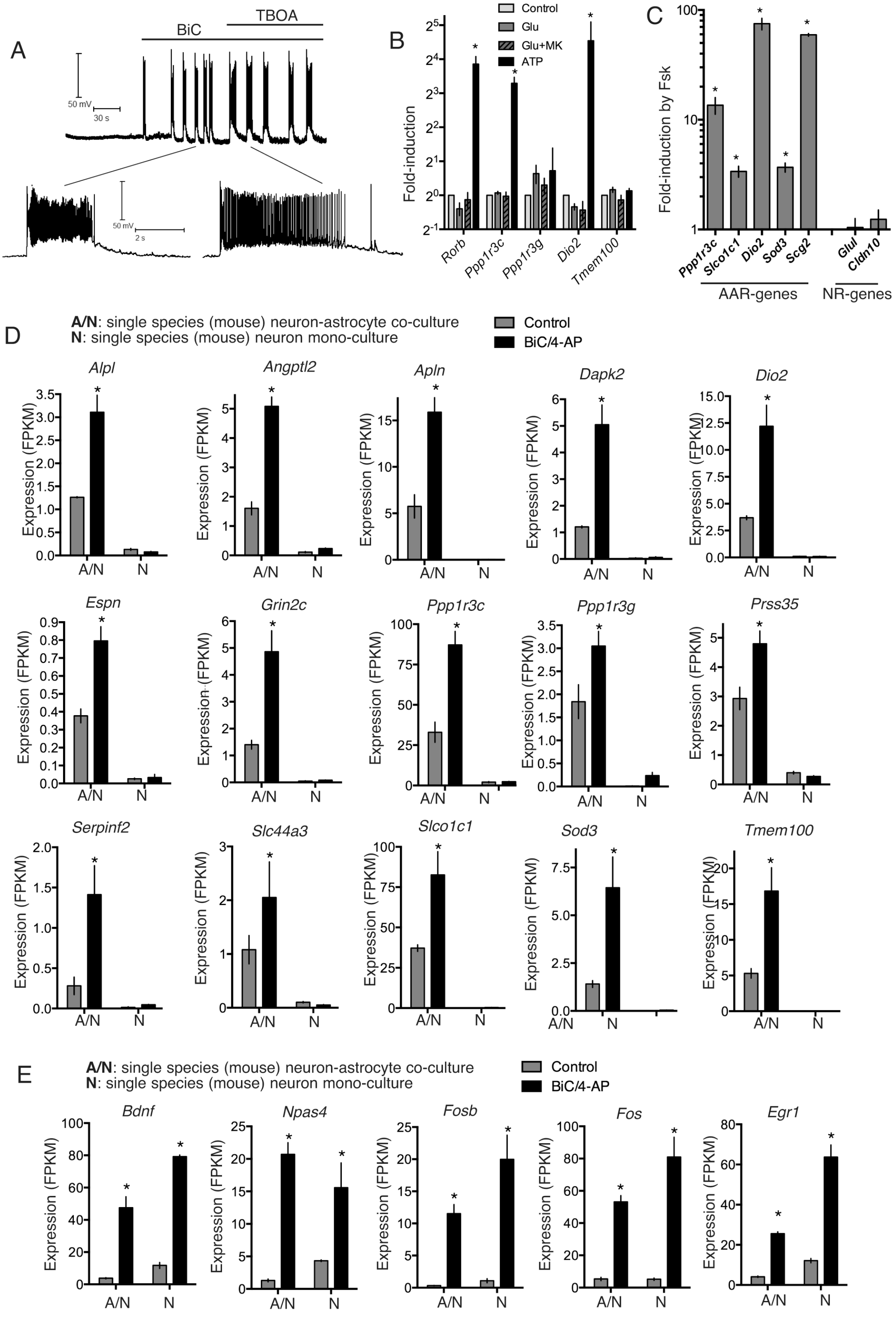

Supplementary Figure 6. A) Example traces illustrating the lengthening of AP bursting upon the addition of TBOA. **B)** Glutamate and NMDAR activation does not influence AAR gene expression, in contrast to ATP. Cultures of mouse astrocytes and rat neurons were treated where indicated with glutamate (20 µM), MK-801 (10 µM), or ATP (100 µM) for 4h (all in the presence of TTX to prevent neuronal firing), after which astrocytic expression of the indicated genes was measured by qPCR using mouse-specific primers. *P<0.05 (2-way ANOVA plus Sidak's post-hoc test, n=3). **C)** Forskolin treatment is sufficient to induce astrocytic activity response (AAR) genes but does not influence neuronally-regulated (NR) genes associated with astrocytic maturation. This experiment was performed exactly as per Fig. 6h, except that pure astrocyte cultures were

used (n=3). **D)** Study of the activity-dependent astrocytic genes whose expression can be tracked in a single-species co-culture. The graph relates to data on 56 genes induced >2-fold by TTX wash-out induced activity in astrocytes (Fig. 5b, Supplementary Data 12). Fold enrichment of these genes in a mouse astrocyte/neuron co-culture compared to a mouse neuronal mono-culture (RNA-seq, n=3) was calculated and the 15 genes that were enriched >10-fold in both cases (indicating that these genes are overwhelmingly expressed in astrocytes in the mouse astrocyte/neuron co-culture) were focused on. For these 15 genes, we show RNA-seq-based data on the influence of 24 h BiC/4-AP-induced synaptic activity on their expression, in both mouse astrocyte/neuron co-cultures (A/N), as well as mouse neuronal mono-cultures (N). Note the large increases shown in the A/N co-cultures, in contrast to the N mono-cultures. *DESeq2 *P*_adj<0.05, compared to control-treated co-cultures. **E)** Data relating to the BiC/4-AP-induced expression of five genes strongly expressed in neurons. These data reveal their strong induction in both A/N co-cultures, as well as N mono-cultures, in contrast to the genes shown in (D). *DESeq2 *P*_adj<0.05.

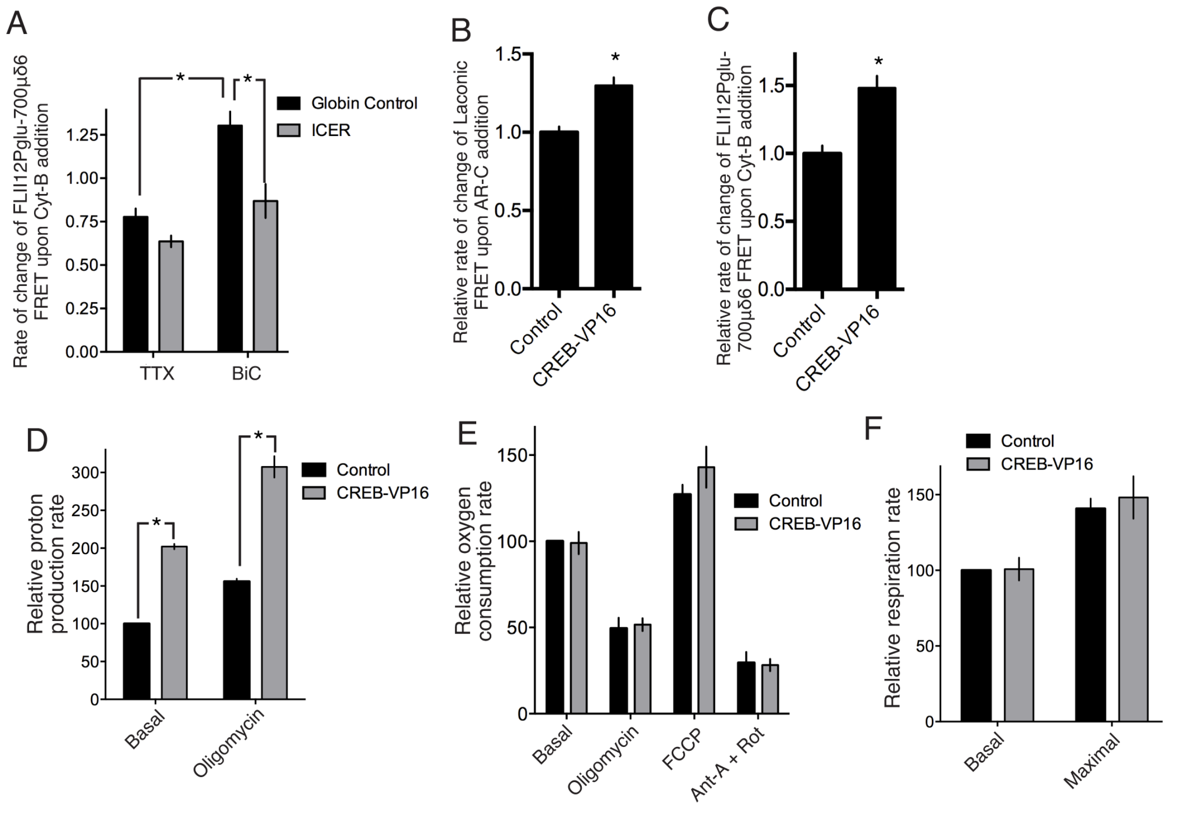

Supplementary Figure 7. A) Activity-dependent CREB-mediated gene expression boosts astrocytic glucose metabolism in human astrocytes. The experiment was performed exactly as in Fig. 7f,g, except that human astrocytes were used instead of mouse astrocytes. P<0.05 (2-way ANOVA plus Sidak's post-hoc test, n=16). **B, C)** CREB-dependent gene expression is sufficient to boost glucose metabolism and lactate export. Astrocytes were transfected with the lactate FRET probe Laconic (B) or the glucose FRET probe LII12Pglu-700µδ6 (C), plus either a control vector (globin) or CREB-VP16 vector. Lactate export and glucose metabolism were analysed exactly as per Figs 7d and 7f respectively. *P<0.05 (t-test, n=25-40 cells per condition per experiment). **D)** CREB-dependent gene expression boosts glycolysis. Astrocytes were infected with control or CREB-VP16 AAV and proton production rate (PPR) measured using a Seahorse Bioanalyser to measure basal glycolysis as well as maximal glycolytic rate exposed by blocking mitochondrial ATP production by oligomycin treatment (1 µg/ml). PPR was normalized to protein levels and expressed as a percentage of basal PPR in control-transfected astrocytes *P<0.05, 2-way ANOVA followed by Sidak's post-hoc test (n=3). **E)** Astrocytes were infected with control or CREB-VP16 AAVs and oxygen consumption rate (OCR) measured under basal conditions, following inhibition of ATP production (oligomycin), mitochondrial uncoupling (FCCP, 2 µM) and inhibition of the electron transport (antimycin A (1 µM) plus rotenone (0.4 µM)). **F)** Basal and maximal respiration rates calculated by taking basal and FCCP-induced OCR and subtracting OCR in the presence of antimycin A plus rotenone, using data shown in (E).
